# Nondestructive Evaluation of Residual Stress in Shot Peened Inconel Using Ultrasonic Minimum Reflection Measurement

**DOI:** 10.3390/ma16145075

**Published:** 2023-07-18

**Authors:** Yeong-Won Choi, Taek-Gyu Lee, Yun-Taek Yeom, Sung-Duk Kwon, Hun-Hee Kim, Kee-Young Lee, Hak-Joon Kim, Sung-Jin Song

**Affiliations:** 1 School of Mechanical Engineering, Sungkyunkwan University, Suwon 16419, Republic of Korea; 0won@skku.edu (Y.-W.C.); hjkim21c@skku.edu (H.-J.K.); sjsong@skku.edu (S.-J.S.); 2 Shalom Engineering Co., Ltd., Hanam 12988, Republic of Korea; ltg@shalomeng.co.kr; 3 Department of Smart Mechanical Components and Materials, Dongyang University, Yeongju 36040, Republic of Korea; 4 Department of Physics, Andong University, Andong 36729, Republic of Korea; sdkwon@anu.ac.kr; 5 Doosan Heavy Industries and Construction Co., Ltd., Changwon 51711, Republic of Korea; hunhee1.kim@doosan.com; 6 KPC Metal Co., Ltd., Gyeongsan 38412, Republic of Korea; kylee@kpccorp.co.kr

**Keywords:** residual stress, shot peening, minimum reflection, INCONEL 718, nondestructive evaluation

## Abstract

Shot peening is a process wherein the surface of a material is impacted by small, spherical metal shots at high velocity to create residual stresses. Nickel-based superalloy is a material with high strength and hardness along with excellent corrosion and fatigue resistance, and it is therefore used in nuclear power plants and aerospace applications. The application of shot peening to INCONEL, a nickel-based superalloy, has been actively researched, and the measurement of residual stresses has been studied as well. Previous studies have used methods such as perforation strain gauge analysis and X-ray diffraction (XRD) to measure residual stress, which can be evaluated with high accuracy, but doing so damages the specimen and involves critical risks to operator safety due to radiation. On the other hand, ultrasonic testing (UT), which utilizes ultrasonic wave, has the advantage of relatively low unit cost and short test time. One UT method, minimum reflection measurement, uses Rayleigh waves to evaluate the properties of material surfaces. Therefore, the present study utilized ultrasonic minimum reflectivity measurements to evaluate the residual stresses in INCONEL specimens. Specifically, this study utilized ultrasonic minimum reflection measurements to evaluate the residual stress in INCONEL 718 specimens. Moreover, an estimation equation was assumed using exponential functions to estimate the residual stress with depth using the obtained data, and an optimization problem was solved to determine it. Finally, to evaluate the estimated residual stress graph, the residual stress of the specimen was measured and compared using the XRD method.

## 1. Introduction

Nuclear power plants are being installed worldwide as a solution to climate change and energy depletion. Inconel, a major material for nuclear power plants and aerospace, is a representative heat-resistant alloy that has excellent strength and hardness compared to other alloys and maintains mechanical properties even at high temperatures. Due to the properties of this material, it is frequently exposed to extreme environments, something which causes material degradation and causes defects. Therefore, shot peening has been implemented and studied for a long time to increase the properties of this material [1,2,3,4].

Shot peening is one of the peening processes wherein high velocity small, spherical metal shots are used to impact the surface of a material with the end goal of plastic de-formation. The deformation generated by this process creates residual stress layers on the surface of the material, a phenomenon which greatly increases the fatigue life and resistance of metal parts, therefore improving overall durability and reliability [5]. Therefore, residual stress evaluation for shot peened material directly related to safety is very important and essential.

Residual stress is typically evaluated through destructive testing and nondestructive testing, and a representative method of destructive testing is perforation strain gauge analysis [6]. Although this method can be used to evaluate residual stress with high accuracy, it damages the specimen. Among methods that measure residual stress using nondestructive testing, the most representative are X-ray diffraction (XRD), Eddy current testing (ECT), and Ultrasonic testing (UT). With XRD, high-accuracy residual stress evaluation can be achieved, but since radiation must be used, it has locational limitations as well as a high inspection unit cost. In the case of ECT, mechanical property evaluation studies on the surface area of materials are being actively conducted, but there is a need for additional research into residual stress measurement [7,8].

Meanwhile, UT has the advantages of low unit price, short inspection time, and enabling the evaluation of not only the surface but also the inside of a material. Among them, Rayleigh waves are one of the ultrasonic wave types used in UT and are mainly used when inspecting the surface of a specimen [9]. When Rayleigh waves propagate along the surface of a specimen, various phenomena such as back-scattered beam, null field, Schoch displacement, and leaky wave are observed on the surface [10,11]. Research has been conducted to evaluate the surface area characteristics of Rayleigh waves with these phenomena [12,13,14,15]. In addition, studies on the dispersion of Rayleigh waves, surface treatment of specimens, and prediction of residual stress using Rayleigh waves have been conducted [16,17], and the measurement of Rayleigh wave velocity has been studied with contact transducers, non-contact sensors, and minimum reflection measurement methods [17,18,19,20,21]. As such, many studies on residual stress evaluation using Rayleigh waves have been conducted nondestructively, but research on residual stress estimation using Rayleigh waves is insufficient and additional research is needed [17,22].

In this paper, in order to predict the residual stress distribution within the specimen surface, Rayleigh wave velocity dispersion data for Inconel 718 specimens were obtained through ultrasonic minimum reflection experiments, and a graph of residual stress distribution in the depth direction of the specimen was estimated using these data. Moreover, an expression consisting of an exponential function was assumed for the estimation, and an optimization problem was defined and solved to determine this assumed expression. Finally, the estimated residual stress distribution profile obtained was compared with the residual stress distribution obtained by depth using X-ray diffraction of the specimen.

## 2. Theory

### 2.1. Characteristics of Residual Stress by Shot Peening

**Figure 1:**
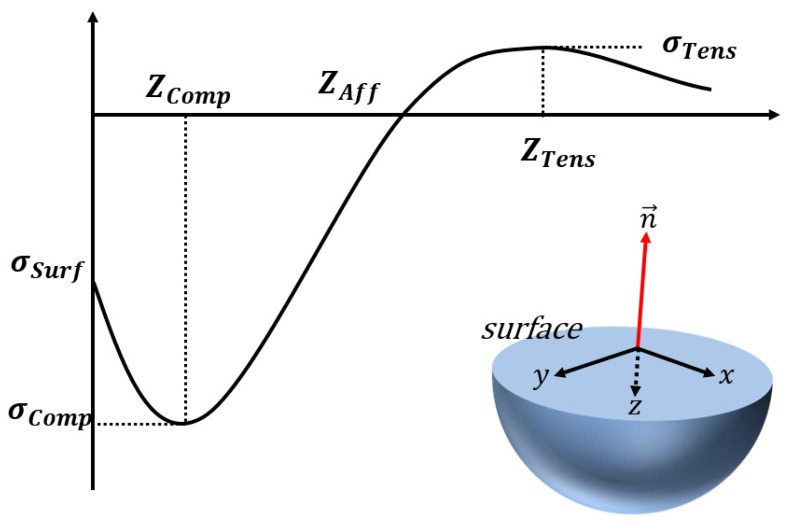
Characteristics of residual stress by shot peening according to depth (the schematization was based on [23]).

The shot peened material surface undergoes various changes, including structural changes in the grain due to plastic deformation, geometric changes due to dimples caused by small shots, and changes in the residual stress profile according to depth [1,22]. As a result of these changes, compression residual stress is formed on the surface, and tensile residual stress occurs under the compression residual stress to maintain equilibrium inside the material. Figure 1 shows the residual stress profile according to the depth direction of the specimen.

Compressive stresses appear at the surface (Z = 0) of the stressed media, maximum compressive residual stress ($\sigma_{COMP}$) occurs at a point slightly deeper than the surface, and compressive residual stresses disappear with increasing depth due to equilibrium, and tensile residual stresses occur.

### 2.2. Minimum Reflection Method and Measurement

**Figure 2:**
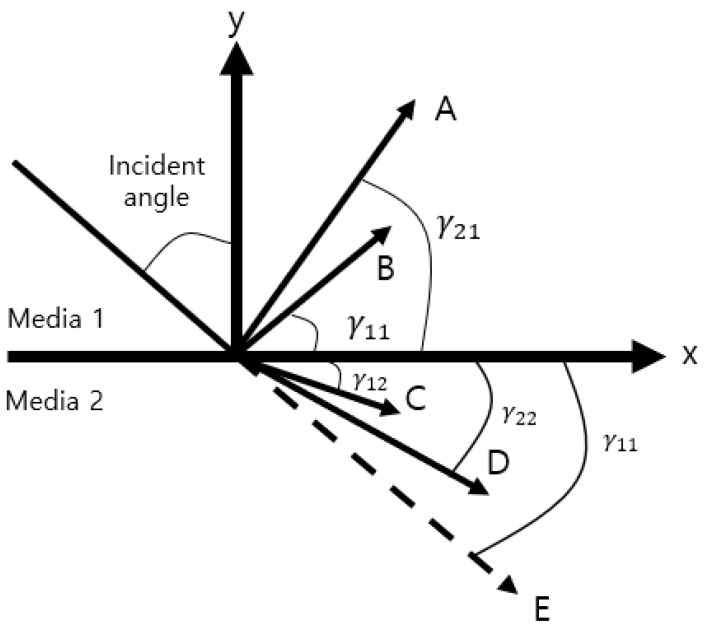
Schematic diagram of a wave incident at an angle to the interface of different media.

Ultrasonic waves that propagate are obliquely generated by a transducer at the interface between media 1 and media 2, where attenuation exists and causes reflection, refraction, and mode conversion at the interface. Figure 2 shows a schematic diagram of the waves that are incident obliquely on the interface of different media.

**Figure 3:**
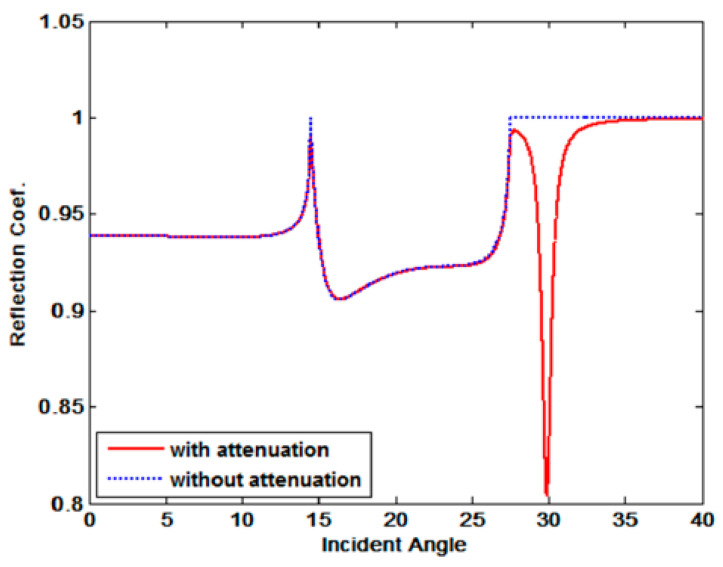
Reflection coefficient with and without attenuation at the water-steel interface [10].

A represents the refracted transverse wave, $\gamma_{21}$ represents the angle, B represents the refracted longitudinal wave, and $\gamma_{11}$ represents the angle. C represents the longitudinal wave transmitted through the specimen, $\gamma_{12}$ represents the angle, D represents the transverse wave transmitted through the specimen, $\gamma_{22}$ represents the angle, E represents the path of the incident wave through the same media (impedance difference = 0), and $\gamma_{11}$ represents the angle. Some of the energy is reflected at the same angle as the incident angle, and the other part of the energy is converted to a Rayleigh wave that propagates along the surface; most of the energy is distributed within one wavelength depth from the surface. As shown in Figure 3, the reflection coefficient at the interface between media 1 and media 2 can be obtained using Equations (1) and (2) as follows [24]:

(1) $$R^{P;P}\left(\omega \right)=\frac{\cos^{2}\;2\gamma_{22}+{\left(\frac{C_{22}}{C_{12}}\right)}^{2}sin2\gamma_{12}sin2\gamma_{22}+\frac{\rho_{1}c_{11}sin\gamma_{12}}{\rho_{1}c_{12}sin\gamma_{11}}}{\cos^{2}\;2\gamma_{22}+{\left(\frac{C_{22}}{C_{12}}\right)}^{2}sin2\gamma_{12}sin2\gamma_{22}-\frac{\rho_{1}c_{11}sin\gamma_{12}}{\rho_{1}c_{12}sin\gamma_{11}}}$$

(2) $$c_{ij}=\frac{v_{ij}}{1-\frac{i\alpha_{ij}v_{ij}}{\omega }}\left(i=1,2\;\;j=1,2\right)$$

where $v_{ij}$ is the ultrasonic velocity of the media i and j, and $\alpha_{ij}$ is the attenuation coefficient of the media i and j. Based on Equations (1) and (2), Figure 3 below shows the reflection coefficient when the attenuation of the specimen exists and the reflection coefficient when the attenuation of the specimen does not exist.

**Figure 4:**
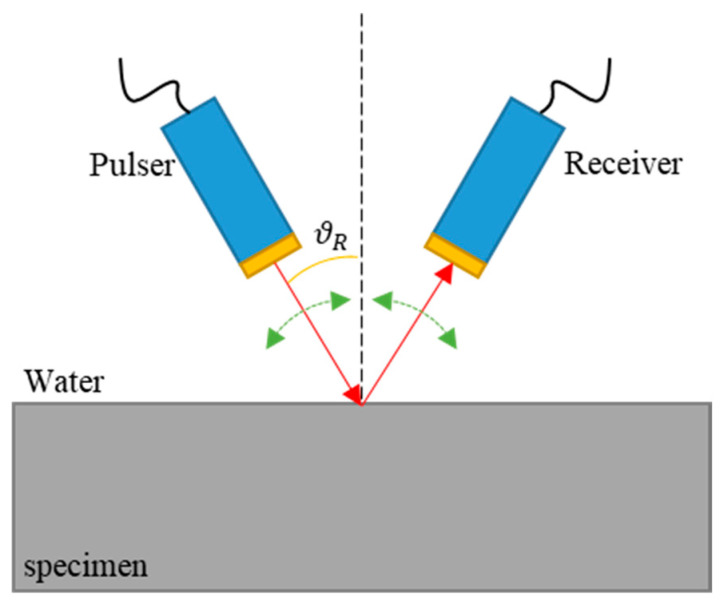
Schematic of an ultrasonic minimum reflection measurement.

Figure 3 is a graph comparing the reflection coefficient (solid line) of the specimen with attenuation and the reflection coefficient of the specimen without attenuation. In the case of a specimen with attenuation, it can be seen that the reflection coefficient decreases rapidly at a specific angle, which is called the minimum reflection, and the angle at that time is the Rayleigh angle ($\theta_{R}$). Therefore, a Rayleigh angle at which a Rayleigh wave is generated occurs in a material in which attenuation exists. The method used to measure the minimum reflection is the pitch–catch setup consisting of a transducer and a receiver, and this method measures the energy change in ultrasonic waves by changing the incident angle. Figure 4 schematically depicts the measurement of the minimum reflection.

### 2.3. Relationship between Minimum Reflection and Rayleigh Wave Velocity

Ultrasonic waves propagated obliquely at the interface between water and specimen cause refraction due to the differences in impedance between the two media. The angle of refraction of the longitudinal and shear wave in the test piece can be obtained by Snell’s law as shown in the following Equation (3):

(3) $$\frac{C_{i}}{sin\;(\theta_{i})}=\frac{C_{L}}{sin\;(\theta_{L})}=\frac{C_{S}}{sin\;(\theta_{S})}$$

where $C_{i}$ is the velocity of the ultrasonic wave in water, $C_{L}$ is the longitudinal wave velocity in the specimen, $C_{s}$ is the shear wave in the specimen, $\theta_{i}$ is the incident angle, $\theta_{L}$ is the longitudinal wave refraction angle, and $\theta_{S}$ is the shear wave refraction angle. When $\theta_{i}$ changes $\theta_{R}$, most of the energy of the incoming ultrasonic wave is transformed into a Rayleigh wave that propagates along the surface without reflection to the specular direction, and the Rayleigh wave travels with a speed that can be obtained using Equation (4):

(4) $$C_{R}=\frac{C_{i}}{sin\;(\theta_{R})}$$

where $C_{R}$ is the Rayleigh wave velocity, which is obtained using Snell’s law.

### 2.4. Relation between Rayleigh Wave Dispersion and Residual Stress Change According to Depth

D. Husson et al. [12] and J. J. Ditri et al. [13] studied the relationship between residual stress and surface waves using the perturbation theory of surface waves. According to the perturbation theory [12,13], the phase shift of the Rayleigh wave which propagates along the surface on the specimen can be calculated using Equation (5):

(5) $$\delta \emptyset =-\frac{\omega }{4P}\int GdV$$

where δ∅ is the phase shift of the Rayleigh wave, ω is the circular frequency, V is the volume of the specimen, ∫G can be expressed by second (λ, μ) and third (l,m,n) order elastic constants of the media along with initial deformation gradients caused by the Rayleigh wave, and P is the average of the power carried per unit width in the direction perpendicular to the direction of propagation of the Rayleigh wave during one time period, which can be expressed as shown in Equation (6):

(6) $$P=\frac{\omega \rho_{0}V_{0}}{2}\left(\frac{\frac{1}{V_{0}^{2}}+K_{s}^{2}}{2K_{s}}-2\frac{\frac{K_{2}}{V_{0}^{2}}+K_{4}K_{s}^{2}}{K_{s}+K_{l}}+\frac{\frac{K_{2}^{2}}{V_{0}^{2}}+K_{4}^{2}K_{s}^{2}}{2K_{l}}\right)\;K_{s}=\sqrt{\frac{1}{V_{0}^{2}}-\frac{1}{V_{s}^{2}}},\;K_{l}=\sqrt{\frac{1}{V_{0}^{2}}-\frac{1}{V_{l}^{2}}}\;K_{2}=\frac{2K_{s}K_{l}}{\left(\frac{1}{V_{0}^{2}}+K_{s}^{2}\right)},\;K_{4}=\frac{2}{\left(1+V_{0}^{2}K_{s}^{2}\right)}$$

where $\rho_{0}$ is the density of the specimen, $V_{0}$ is the velocity of the Rayleigh wave, and $V_{s}$ and $V_{l}$ are transverse wave and longitudinal wave velocity, respectively, corresponding to the unstressed media.

G in Equation (5) can be expressed as $a_{i}$ and $b_{i}$ $\left(i\in \left\{1,2,3\right\}\right)$ as follows [13]:

(7) $$\begin{array}{ll} G\equiv \frac{\partial b_{m}}{\partial a_{m}} & \left\{\left(2l+\lambda \right)\left[A\left(a_{2},w\right)+B\left(a_{2},w\right)+C\left(a_{2},w\right)\right]+\left(\lambda +m\right)D\left(a_{2},w\right)+mE\left(a_{2},w\right)\right\}\\ & \;\;+\frac{\partial b_{2}}{\partial a_{2}}\left\{\left(2\lambda +6\mu +4m\right)A\left(a_{2},w\right)+\mu \left[2D\left(a_{2},w\right)+E\left(a_{2},w\right)\right]\right\}\\ & \;\;+\frac{\partial b_{3}}{\partial a_{3}}\left\{\left(2\lambda +6\mu +4m\right)B\left(a_{2},w\right)+\mu \left[2D\left(a_{2},w\right)+E\left(a_{2},w\right)\right]\right\}\\ & \;\;-\frac{\partial b_{1}}{\partial a_{1}}\{\left(\frac{n}{2}\right)\left[D\left(a_{2},w\right)+E\left(a_{2},w\right)\right]+\left(\lambda +2m-n\right)C\left(a_{2},w\right)\} \end{array}$$

where functions $A\left(a_{2},w\right)$ through $E\left(a_{2},w\right)$ depend upon the gradients of the displacement field of the Rayleigh wave that propagates on the unstressed media. The distribution profile of residual stress along the depth of the uniform field can be represented as a function of depth as $\sigma_{33}(a_{2})$, as was proposed by J. J. Ditri [13]. Equation (5), therefore, can be expressed as Equations (8) and (9) [13]:

(8) $$\delta \emptyset^{33}(\omega )=-\frac{L_{0}\omega }{4P}\int_{0}^{\infty }\alpha_{i}^{∥}F_{i}\left(a_{2},\omega \right)\sigma_{33}\left(a_{2}\right)da_{2}$$

(9) $$F_{i}\left(a_{2},\omega \right)=\omega^{2}\{f_{i1}e^{-2\omega K_{s}a_{2}}+f_{i2}e^{-2\omega K_{l}a_{2}}+f_{i3}e^{-\omega (K_{l}+K_{s})a_{2}}\}$$

where $f_{ij},$ according to perturbation theory [13], as well as index i∈{1,…,5} and j∈{1,2,3}, are given as follows:

$$f_{11}={\left(\frac{K_{s}}{V_{0}}\right)}^{2},\;\;\;\;f_{12}={\left(\frac{K_{1}K_{2}}{V_{0}}\right)}^{2},\;\;\;\;f_{13}=-\frac{2K_{S}{K_{l}}_{K_{2}}}{V_{0}^{2}}\;f_{21}=f_{11};\;\;f_{22}=K_{4}^{2}f_{21},\;\;f_{23}=-2K_{4}f_{21}\;f_{31}=-2f_{11};\;\;f_{32}=\frac{K_{l}K_{2}K_{4}f_{31}}{K_{s}},\;\;f_{33}=-\left[K_{4}+\frac{K_{l}K_{2}}{K_{s}}\right]f_{31}\;f_{41}=\frac{1}{V_{0}^{4}}+K_{s}^{4};\;\;f_{42}=\frac{K_{2}^{2}}{V_{0}^{4}}+{\left(K_{s}K_{l}K_{4}\right)}^{2},\;\;f_{43}=-2[\frac{K_{2}}{V_{0}^{4}}+K_{s}^{3}K_{l}K_{4}]\;f_{51}=2f_{11};\;\;f_{52}=\frac{K_{l}K_{2}K_{4}f_{51}}{K_{s}},\;\;f_{53}=-\left[\frac{K_{l}K_{4}}{K_{s}}+K_{2}\right]f_{51}$$

According to the perturbation theory [13], the constants $\alpha_{i}^{∥}$ in Equation (8) are given as follows:

$$\alpha_{1}^{∥}\equiv \frac{1}{\left(3\lambda +2\mu \right)}\{\lambda +2l-\frac{\lambda (2\lambda +6\mu +4m)}{2\mu }\}\;\alpha_{2}^{∥}\equiv \frac{1}{\left(3\lambda +2\mu \right)}\{\lambda +2l-\frac{\left(\lambda +\mu )(2\lambda +6\mu +4m\right)}{\mu }\}\;\alpha_{3}^{∥}\equiv \frac{1}{\left(3\lambda +2\mu \right)}\left\{\lambda +2l-\frac{\lambda \left(\lambda +2m-n\right)}{2\mu }\right\}\;\alpha_{4}^{∥}\equiv \frac{1}{\left(3\lambda +2\mu \right)}\left\{3\lambda +2\mu +m-\frac{\lambda \left(2\mu -\frac{n}{2}\right)}{2\mu }\right\}\;\alpha_{5}^{∥}\equiv \frac{1}{\left(3\lambda +2\mu \right)}\left\{\lambda +\mu +m-\frac{\lambda \left(\mu -\frac{n}{2}\right)}{2\mu }\right\}$$

Therefore, the results for the change in phase and the propagation in the direction parallel to the specimen surface can be converted to change in phase velocity using the following Equation (9):

(10) $$\frac{\Delta V}{V_{0}}=\bar{\varepsilon_{33}}-\frac{\delta \emptyset^{33}V_{0}}{\omega L_{0}}$$

where $\bar{\varepsilon_{33}}$ denotes the surface strain in the direction of propagation and $\frac{\Delta V}{V_{0}}$ denotes the relative change in phase velocity of the surface wave propagating along the surface of the stressed media [13].

Using Equation (10), an inversion can be performed to estimate the residual stress $\sigma_{33}\left(a_{2}\right)$ on the media. To that end, to describe the major characteristics of the residual stress distribution along the depth, any kind of suitable curve fitting approach can be used. The efficiency and the accuracy of such an inversion would be dependent on the adopted curve fitting approach.

## 3. Ultrasonic Minimum Reflection Measurement

**Figure 5:**
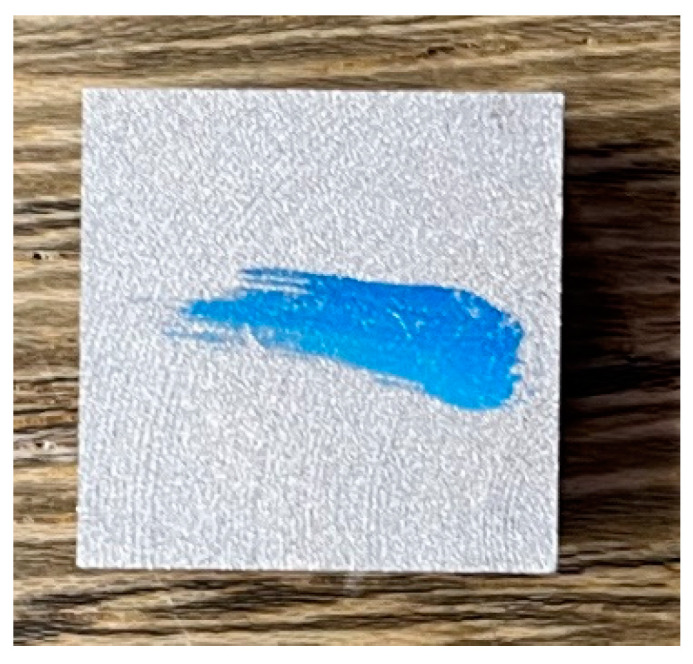
INCONEL 718 specimen.

Figure 5 shows the test specimen, which is made of the material Inconel 718, with shot peening. The specimen’s dimensions are 20 mm (width) × 20 mm (depth) × 15 mm (height).

**Figure 6:**
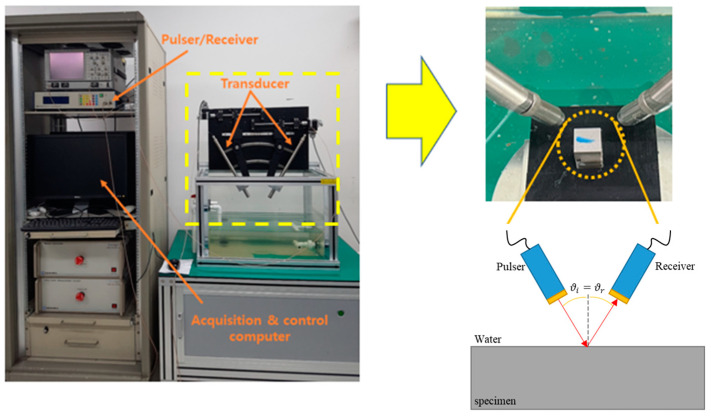
Ultrasonic immersion testing experiment setup with pitch-catch method.

Figure 6 shows the ultrasonic minimum reflection measurement setup.

**Figure 7:**
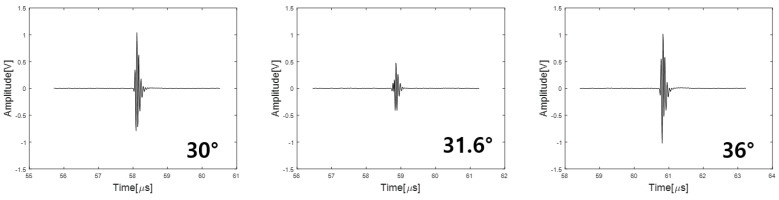
A-scan data acquired in the specific angle range.

The measurement system equipment is composed of a pulser/receiver, a pair of transducers for a pitch–catch measurement, and a data acquisition and control computer. The transducers are planar and circular with a center frequency of 20 MHz and a diameter of 0.25″. During the measurement, the incident angles were changed from 30 degrees to 36 degrees with the step of 0.02 degrees. The data acquisition software was home-made using the LabVIEW 2021 program from National Instrument. We acquired A-scan data in a specific angle range and Figure 7 shows the data obtained at a specific angle.

**Figure 8:**
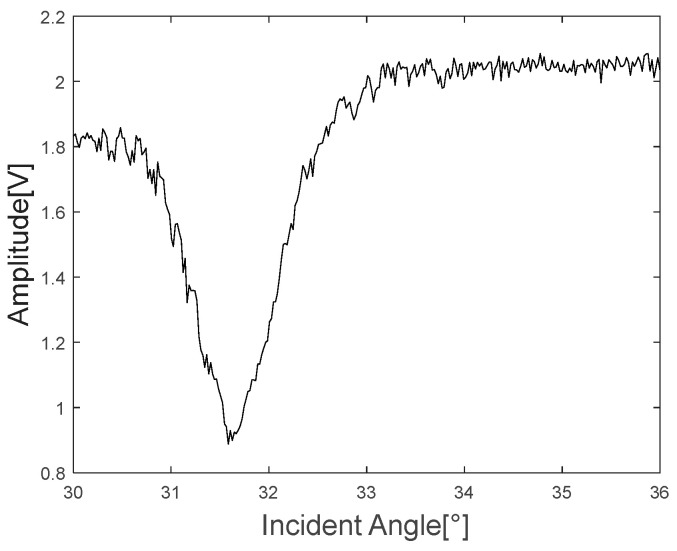
Minimum reflection profile.

Figure 7 shows the A-scan at the beginning (30 degrees) and end of the angle range (36 degrees), as well as the angle (31.6 degrees) at which the lowest A-scan signal was obtained. Figure 8 shows the minimum reflection profile with each peak-to-peak of 300 A-scan data.

**Figure 9:**
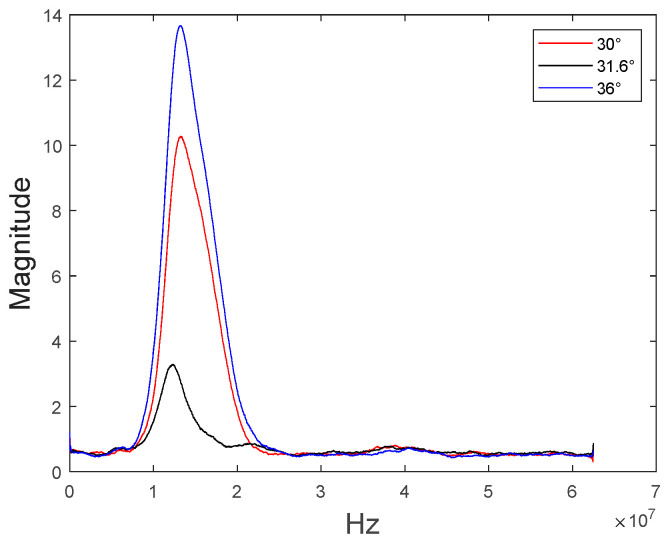
Minimum reflection frequency spectrum.

As shown in Figure 8, the peak-to-peak voltage decreases rapidly at a specific incident angle, and it can also be confirmed that the peak-to-peak voltage is lowest at 31.6 degrees, which is determined by the Rayleigh angle of the specimen. Figure 9 shows the minimum reflection frequency spectrum at each of the three angles.

Figure 9 shows the result of performing Fast Fourier Transform on representative A-scan data. At 30 degrees and 36 degrees, there is little difference in the magnitude at the center frequency of 17 MHz, but at the Rayleigh angle of 31.6 degrees, it can be seen that the magnitude value at the center frequency decreases rapidly. This is attributable to the fact that most of the energy of the incident angle is converted into a Rayleigh wave at the Rayleigh angle.

## 4. Rayleigh Wave Dispersion Determination

**Figure 10:**
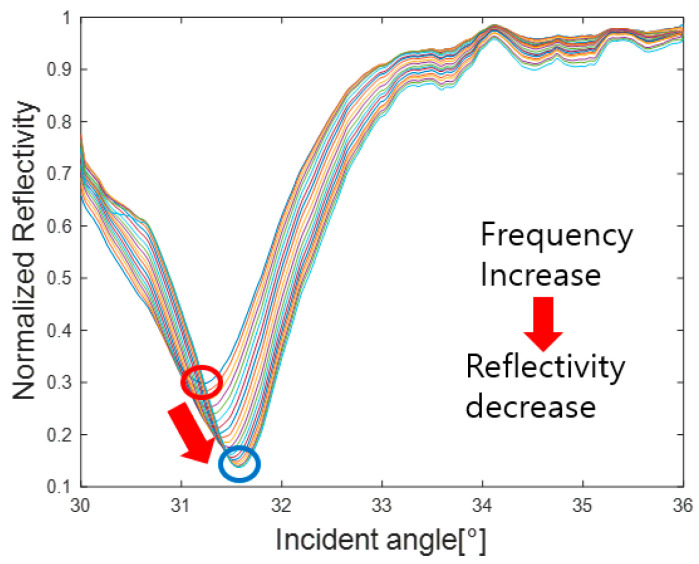
Minimum reflection profiles within the frequency range.

In the next step, the specific frequency band from 11 MHz to 18.5 MHz was selected using the 6 dB drop method. Figure 10 shows a total of 20 standardized minimum reflection profiles from 11 MHz to 18.5 MHz in the selected frequency range.

**Figure 11:**
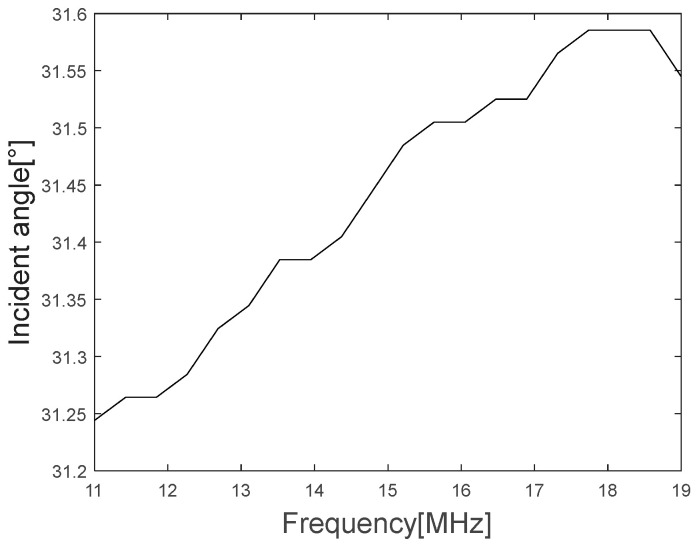
Rayleigh angle variation according to the frequency range.

In Figure 10, the upper red circle represents the Rayleigh angle point at 11 MHz, while the lower blue circle represents the Rayleigh angle point at 18.5 MHz. As shown in Figure 10, it can be confirmed that the Rayleigh angle increases and the reflectivity decreases as the frequency increases, while Figure 11 shows the graph of the proportional relationship.

**Figure 12:**
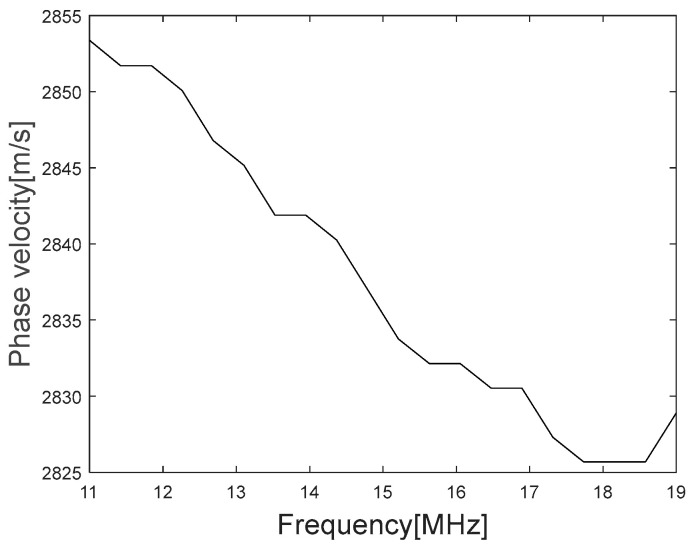
Rayleigh wave dispersion according to the frequency range.

The graph of the Rayleigh angle change according to the frequency can be obtained as the phase velocity of the Rayleigh wave according to Equation (4), and Figure 12 shows the graph of the phase velocity of the Rayleigh wave according to the frequency.

## 5. Inverse Analysis

One of the effective curve fitting approaches is in the combination of two exponential functions, as shown in Equation (11):

(11) $$\sigma_{33}\left(a_{2}\right)=b\left(1\right)e^{b\left(2\right)a_{2}}+b\left(3\right)e^{b\left(4\right)a_{2}}$$

where $b\left(1\right),\;b\left(2\right),\;b\left(3\right),$ and b(4) are undefined coefficients. It is organized as a function for w where the coefficients exist. Using Equation (11), the analytical evaluation of integrals in Equation (8) can be performed more effectively. Furthermore, when using Equation (11), it becomes easier to get maximum compression stress in the residual stress graph. For inverse analysis, the values of the variables in Equation (10) are listed in Table 1 [25].

**Figure 13:**
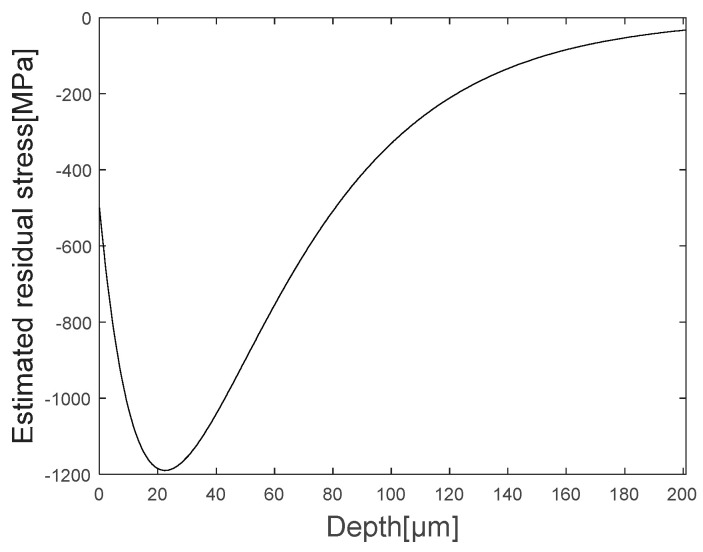
Estimated residual stress profile by solved optimization problem.

Substituting Equation (8) into Equation (10), and then using Equation (11) and the assumptions in Table 1, the equation of w can be obtained with the coefficients as follows:

(12) $$\frac{\Delta V\left(w\right)}{V}-\bar{\varepsilon_{33}}=0.082w^{3}(\frac{{1.9*10}^{-14}b\left(1\right)}{1.86*10^{-4}w-b\left(2\right)}+\frac{{1.9*10}^{-14}b\left(3\right)}{1.86*10^{-4}w-b\left(4\right)}+\frac{4.07*10^{-14}b\left(1\right)}{6.28*10^{-4}w-b\left(2\right)}+\frac{4.07*10^{-14}b\left(3\right)}{6.28*10^{-4}w-b\left(4\right)}+\frac{-4.8*10^{-14}b\left(1\right)}{4.07*10^{-4}w-b\left(2\right)}+\;\frac{-4.8*10^{-14}b\left(3\right)}{4.07*10^{-4}w-b\left(4\right)}$$

where the left term is obtained from the data shown in Figure 12 and the right term is the function to be optimized using the nonlinear optimization. The optimization is solved by the problem using the nonlinear least square method, which is a technique that estimates model parameters by minimizing the sum of the squares of the residuals between the obtained data and model (the right terms) and which is sensitive to the initial values of the parameters [26]. Therefore, to solve the optimization problem, an objective function is defined by Equation (12), the initial values were carefully chosen, and $\bar{\varepsilon_{33}}$ was excluded for being a constant. Moreover, constraints were set to satisfy the physical phenomenon of the stressed specimen mentioned in Section 2.1. By solving the optimization problem where the objective function is minimized, the estimated residual stress profile shown in Figure 13 is obtained using Equation (11).

## 6. Comparison with XRD Measurement

**Figure 14:**
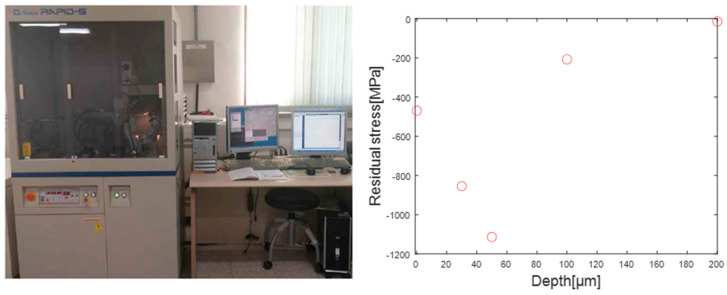
XRD equipment for residual stress measurement of the specimen [4].

To compare the estimated residual stress profile with the actual residual stress values, XRD measurements were performed on the specimens. The specimen was measured using the equipment shown on the left side of Figure 14, and the results obtained at five different depths (x = 0 μm, 25 μm, 50 μm, 100 μm, 200 μm) are shown on the right side of the same figure.

**Figure 15:**
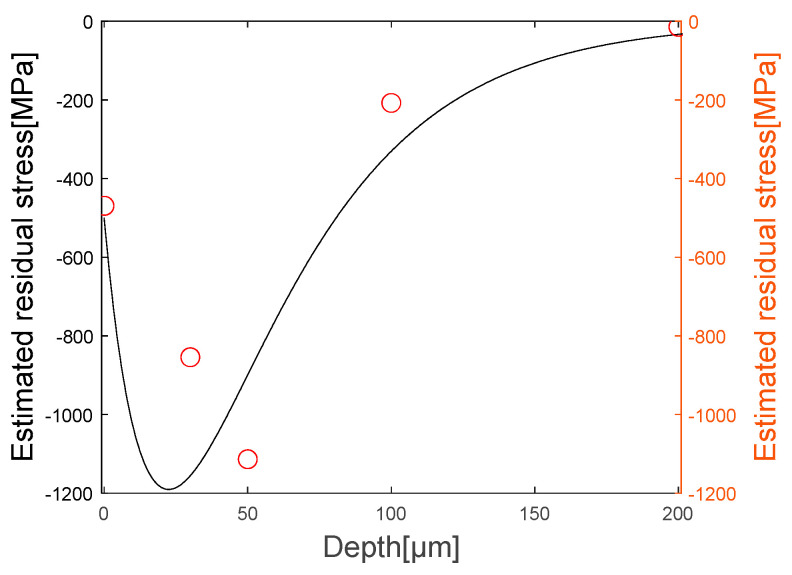
Comparison of experiment results and XRD results.

Similar to the physical phenomenon mentioned earlier, it can be seen that compressive residual stress exists at the surface on the specimen, and that the compressive residual stress disappears as the depth increases after the maximum compressive residual stress. Figure 15 is a comparison between the estimated graph and the obtained graph, and it can be confirmed that the estimated graph has a similar shape of residual stress distribution and maximum compressive stress value of the measured specimen.

## 7. Conclusions

In this study, a robust method was developed to nondestructively estimate the residual stress distribution along the depth direction of a shot peened specimen from the Rayleigh wave dispersion data.

For that purpose, first, the Rayleigh wave velocity was measured at different incident angles by using the ultrasonic minimum reflection measurement in a pitch–catch setup with the peened specimen immersed in water. Second, the velocity dispersion of the Rayleigh wave was obtained by applying a frequency analysis to the experimentally measured ultrasonic reflection data.

Third, the residual stress distribution along the depth direction was estimated from these Rayleigh wave dispersion data. For doing that, a relationship, proposed by Ditri and Hongerholt [13], between Rayleigh wave dispersion and residual stress variation was considered in this study. In addition, the residual stress distribution was assumed to be described by a powerful combination of two simple exponential functions. As a result, a nonlinear optimization problem was formulated. Finally, this nonlinear optimization problem was solved with carefully chosen initial values of the parameters in the assumed exponential functions.

From the analyses described above, the residual stress distribution along the depth of the specimen was successfully estimated with a very good agreement with the residual stress measured by X-ray diffraction method. This good agreement demonstrates the robustness of this approach as a nondestructive quality control tool for estimating residual stress distribution in shot-peened parts in many industrial applications.

## Figures and Tables

**Table 1:** Assumed values for variables.

Variable (Symbol)	Assumption Value	Variable (Symbol)	Assumption Value
$V_{0}$	2800 [m/s]	m	−606 [GPa]
$V_{l}$	5900 [m/s]	n	−479 [GPa]
$V_{s}$	2900 [m/s]	μ	80 [GPa]
l	−527 [GPa]	λ	121 [GPa]

## Data Availability

The data that support the finding of this study ae available from the corresponding author upon reasonable request.
